# Protocol for a multicenter, double-blind, randomized, placebo-controlled phase III trial of the inhaled β2-adrenergic receptor agonist salbutamol for transient tachypnea of the newborn (the REFSAL trial)

**DOI:** 10.3389/fped.2022.1060843

**Published:** 2023-01-12

**Authors:** Dariusz Madajczak, Thierry Daboval, Ryszard Lauterbach, Beata Łoniewska, Witold Błaż, Tomasz Szczapa, Iwona Sadowska-Krawczenko, Marzena Michalak-Kloc, Helena Sławska, Maria Borszewska-Kornacka, Renata Bokiniec, the REFSAL Study Group

**Affiliations:** ^1^ Department of Neonatology and Neonatal Intensive Care, Medical University of Warsaw, Warsaw, Poland; ^2^ Department of Pediatrics – Division of Neonatology, Faculty of Medicine, University of Ottawa, Ottawa, ON, Canada; ^3^ Neonatology Clinic, University Hospital, Krakow, Poland; ^4^ Department of Neonatology and Intensive Neonatal Care, Pomeranian Medical University, Szczecin, Poland; ^5^ Clinical Department of Neonatology With Neonatal Intensive Care Unit, University of Rzeszow, Saint Jadwiga the Queen Clinical Provincial Hospital No 2, Rzeszow, Poland; ^6^ Department of Neonatology, Poznań University of Medical Sciences, Poznań, Poland; ^7^ Department of Neonatology, Collegium Medicum in Bydgoszcz, Nicolaus Copernicus University, Toruń, Poland; ^8^ Neonatology Clinical Department, Karol Marcinkowski University Hospital, Zielona Góra, Poland; ^9^ Neonatology Unit, Specialist Hospital No 2, Bytom, Medical University of Silesia, Bytom, Poland

**Keywords:** transient tachypnea, respiratory distress, persistent pulmonary hypertension, neonate, salbutamol

## Abstract

**Background:**

Transient tachypnea of the newborn (TTN), which results from inadequate absorption of fetal lung fluid, is the most common cause of neonatal respiratory distress. Stimulation of β-adrenergic receptors enhances alveolar fluid absorption. Therefore, the β2-adrenergic receptor agonist salbutamol has been proposed as a treatment for TTN. This study aims to evaluate the efficacy and safety of salbutamol as supportive pharmacotherapy together with non-invasive nasal continuous positive airway pressure (NIV/nCPAP) for the prevention of persistent pulmonary hypertension of the newborn (PPHN) in infants with TTN.

**Methods and analysis:**

This multicenter, double-blind, phase III trial will include infants with a gestational age between 32 and 42 weeks who are affected by respiratory disorders and treated in eight neonatal intensive care units in Poland. A total of 608 infants within 24 h after birth will be enrolled and randomly assigned (1:1) to receive nebulized salbutamol with NIV or placebo (nebulized 0.9% NaCl) with NIV. The primary outcome is the percentage of infants with TTN who develop PPHN. The secondary outcomes are the severity of respiratory distress (assessed with the modified TTN Silverman score), frequency of need for intubation, duration of NIV and hospitalization, acid–base balance (blood pH, partial pressure of O_2_ and CO_2_, and base excess), and blood serum ionogram for Na^+^, K^+^, and Ca^2+^.

**Discussion:**

The Respiratory Failure with Salbutamol (REFSAL) study will be the first clinical trial to evaluate the efficacy and safety of salbutamol in the prevention of persistent pulmonary hypertension in newborns with tachypnea, and will improve short term outcomes. If successful, the study will demonstrate the feasibility of early intervention with NIV/nCPAP together with nebulized salbutamol in the management of TTN.

**Ethics and dissemination:**

The study protocol was approved by the Bioethics Committee of the Medical University of Warsaw, Warsaw, Poland on November 16, 2020 (decision number KB/190/2020). All procedures will follow the principles of the Declaration of Helsinki. The results of the study will be submitted for knowledge translation in peer-reviewed journals and presented at national and international pediatric society conferences.

**Clinical Trial Registration:**

It is registered at ClinicalTrials.gov NCT05527704, EudraCT 2020-003913-36; Protocol version 5.0 from 04/01/2022.

## 1. Introduction

Transient tachypnea of the newborn (TTN) is the most common cause of neonatal respiratory distress. It affects up to 1% of term, 3.5% of late preterm, and more than 10% of preterm infants born at a gestational age of 33–34 weeks (1, 2). TTN results from inadequate absorption of fetal lung fluid (3). Fetal lung fluid is produced by the developing lung epithelium as a result of the active secretion of chloride ions (Cl^−^) and concomitant passive flow of sodium ions (Na^+^) and water into the fetal alveolar space (4). Physiologically, fetal epinephrine produced during labor that acts *via* β2-adrenergic receptors activates Na^+^ channels and triggers fluid absorption (5). The absence of adequate stress or hormonal function, which decreases Na^+^ reabsorption and results in the retention of fetal lung fluid, has been implicated in the pathophysiology of TTN (6). Stimulation of β-adrenergic receptors by β_2_-adrenergic receptor agonists upregulates alveolar epithelial Na^+^ transport (7) and enhances alveolar fluid absorption (8).

The clinical signs and chest radiograph or chest x-ray (CXR) are crucial in the traditional diagnosis of TTN (9). Unfortunately, CXR is a relatively poor imaging technique. Many studies have criticized the poor quality of x-ray images and discrepancies in the exposure parameters (10). Moreover, CXR involves exposing patients to radiation (11). However, with technological progress and the widespread use of digital techniques in radiological diagnostics, the radiation dose required to fix the CXR images is significantly lower than that for “classic” x-ray images using film.

Nevertheless, a newborn baby may have to get several x-ray images taken during hospitalization, which significantly affects the radiation dose. A newborn's high rate of cell division means that such exposure may increase their risk of cancer by two to three times that of the adults receiving a comparable dose of radiation (10). The risk also increases inversely with a child's immaturity. Lung ultrasound (LUS), which does not have radiation risk, is a repetitive bedside instrument. Therefore, it appears to be an ideal choice for the differential diagnosis of respiratory disorders in newborns.

Utilization of LUS in diagnosing lung diseases has gained increasing attention in recent years. At least a few clinical trials have assessed the usefulness of this method in critically ill adults and newborns. Ma et al. (12) and Szymanski et al. (13) showed that the use of LUS in the diagnosis of TTN, compared to chest radiographs, significantly improves the results and justifies its clinical use.

Although TTN is generally considered benign and self-limiting, it can persist and develop into severe conditions requiring respiratory support, such as persistent pulmonary hypertension of the newborn (PPHN) (14, 15). PPHN affects approximately two per 1,000 live births (16, 17). It is a major cause of neonatal death, with a 4%–33% mortality rate (16). Affected individuals often develop long-term impairments such as neurodevelopmental, cognitive, and hearing abnormalities (18). The syndrome is characterized by sustained elevation of pulmonary vascular resistance, leading to extrapulmonary blood shunting from right to left across persistent fetal channels (patent ductus arteriosus and patent foramen ovale), which can cause severe life-threatening hypoxemia and circulatory failure (19). PPHN is diagnosed in both term and preterm infants, and its etiology includes, in addition to TTN, sepsis, meconium aspiration syndrome, congenital diaphragmatic hernia, congenital pneumonia, birth asphyxia, and respiratory distress syndrome (17). Management of PPHN includes sedation, assisted ventilation, surfactants, pulmonary vasodilators, and inotropic agents (20).

Recommendations for the treatment of TTN include non-invasive oxygen support, preferably in an oxygen therapy (accompanied by routine intensive care activities such as continuous cardiopulmonary monitoring, maintenance of a neutral thermal environment, blood glucose checks, and observation for sepsis). However, continuous positive airway pressure (CPAP) has also proven useful in the prophylaxis and management of TTN (21–23).

The routine use of supportive pharmacotherapy in infants with TTN is poorly documented (24). Given the pathophysiology of fetal alveolar fluid retention in neonates with TTN, furosemide, racemic epinephrine, and salbutamol have been studied in the management of TTN (24). A systematic review of the routine use of diuretics in infants with TTN showed that neither oral nor intravenous furosemide was beneficial with respect to the duration of respiratory symptoms or length of hospitalization (25). A pilot study of nebulized racemic epinephrine in neonates with TTN provided no evidence of efficacy (26, 27).

Studies on salbutamol have demonstrated that this β_2_-adrenergic receptor agonist is effective in the treatment of infants with TTN (14, 15, 28, 29). The study by Talaat et al. (29) on 100 infants with TTN showed that nebulized salbutamol reduced the duration of respiratory support and hospitalization. Several clinical parameters improved significantly, and no side effects were associated with the treatment (29). Nonetheless, a recently published systematic review showed limited evidence that nebulized salbutamol reduces the duration of oxygen therapy for TTN, duration of tachypnea, or need for CPAP and mechanical ventilation (30). Summarizing, the possible benefits and safety of salbutamol in infants with TTN have not yet been fully confirmed. To the best of our knowledge, this will be the first study to examine the potential of early intervention with salbutamol to reduce the risk of PPHN (30).

We propose a research protocol to evaluate the efficacy and safety of salbutamol as supportive pharmacotherapy in association with noninvasive nasal ventilation (NIV) to prevent PPHN in infants with TTN.

## 2. Methods and analysis

### 2.1. Hypothesis

We hypothesize that the early use of inhaled salbutamol in addition to nCPAP in neonates diagnosed with TTN prevents the development of PPHN and need for invasive ventilation.

### 2.2. Trial design

This study is designed as a prospective multicenter, double-blind, randomized, placebo-controlled phase III trial. It will include neonates (gestational age of 32–42 weeks) treated in Poland's eight level 2 and 3 neonatal intensive care units.

Parents of neonates diagnosed with TTN within the first 24 h of life will be offered participation in the study. After providing oral and written information about the study, we will ask both parents to provide written consent for the participation of the neonate in the trial.

We have planned to recruit for the study for 24 months, from December 31, 2021 to December 31, 2023.

### 2.3. Participants, enrolment, randomization, and statistical methods

#### 2.3.1. Randomization

After obtaining signed consent from the parents of the neonates for their participation, the participants will be registered and randomized in an electronic research database.

**Figure 1:**
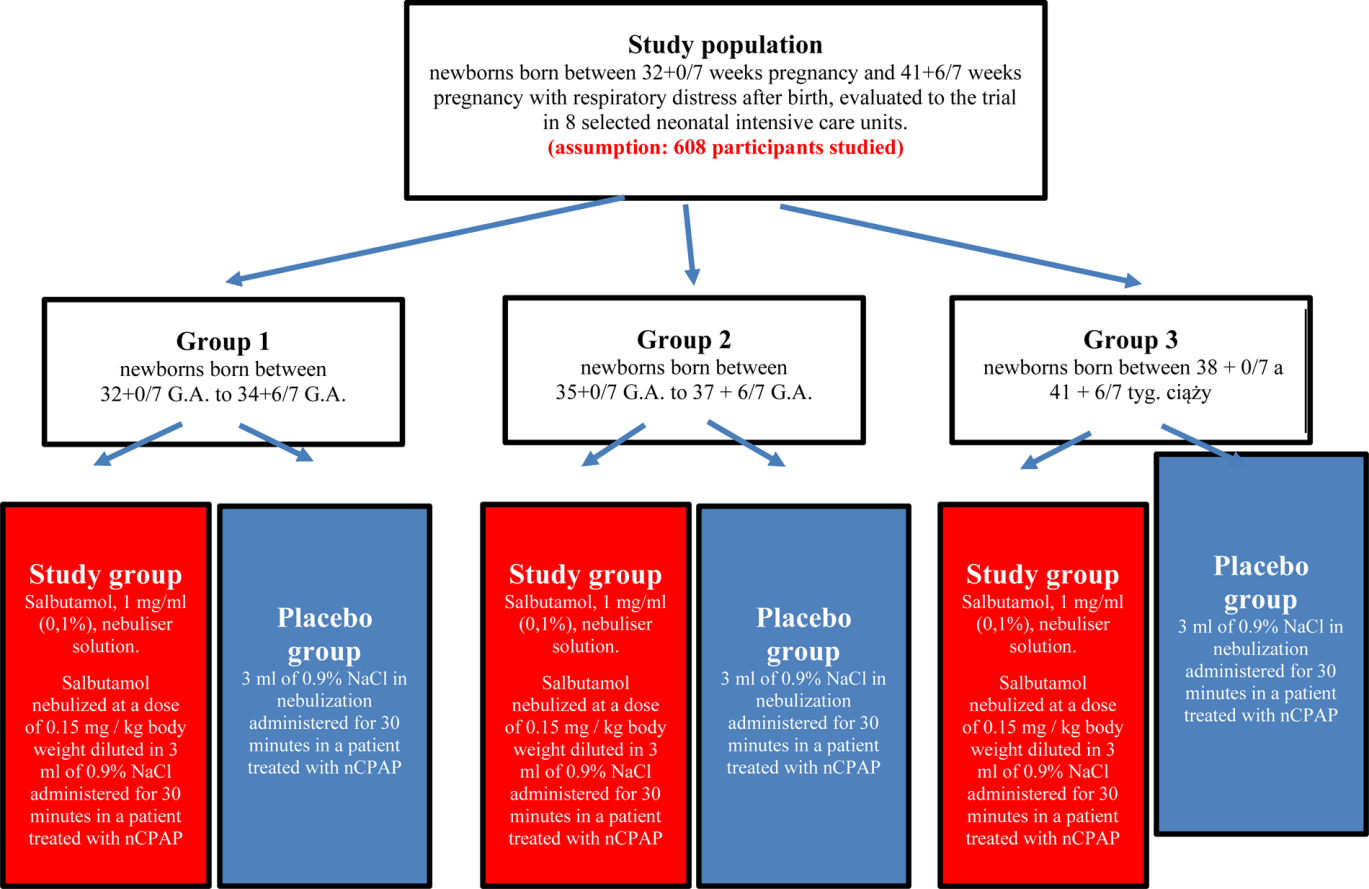
Characteristics of the study group and interventions’ description.

Enrolled infants will be randomly assigned (1:1) to receive nebulized salbutamol with NIV or placebo (nebulized 0.9% NaCl) with NIV. Stratified (according to gestational age: 32 + 0/7 to 34 + 6/7 weeks, 35 + 0/7 to 37 + 6/7 weeks, and 38 + 0/7 to 41 + 6/7 weeks) blocked randomization will be used to ensure homogeneity across treatment groups and sites (Figure 1).

#### 2.3.2. Sample size

A sample size of 506 patients (253 in each group) was estimated to provide 80% power at *α *= 0.05 to detect a difference in the incidence of PPHN between groups, assuming an incidence of 0.1% in the salbutamol group vs. 4% in the placebo group. Assuming a 20% dropout rate (due to the decision of the principal investigator, withdrawal of consent from a parent/legal guardian, or other reasons described in the subsection “Early termination”), the sample size was fixed at 608 patients.

#### 2.3.3. Computational methods

Descriptive statistics for categorical variables will be presented as numbers, percentages, and relative frequencies, and that for continuous variables as means, standard deviations, medians, ranges, and interquartile ranges. The incidence of PPHN (primary outcome) between the salbutamol and placebo groups will be compared using the chi-square or Fisher's exact test (dependent on observed incidence). Potential determinants of PPHN will be analyzed using a logistic regression method. The secondary endpoint TTN scale will be analyzed using the Mann-Whitney test. Other exploratory analyses will be conducted using the Student's or Mann-Whitney *U* tests for continuous variables and the chi-square or Fisher's exact tests for categorical variables. Multivariate analyses will be conducted using linear or logistic regression methods. The normality of the distribution of the analyzed variables will be assessed using the Shapiro-Wilk test. Further, analyses will be performed using R 4.0.2 (or later) statistical software [R Core Team (2021). R: A language and environment for statistical computing. R Foundation for Statistical Computing, Vienna, Austria. URL: https://www.R-project.org/]. In all analyses, *α *= 0.05 will be set as the level of significance.

#### 2.3.4. Inclusion criteria

The inclusion criteria are as follows:

a. Gestational age at birth between 32 and 42 weeks.
b. Respiratory disorders (tachypnea and expiratory grunting) lasting longer than 15 min after birth, present for at least 15 min in the first 6 h of life, or a need for noninvasive respiratory support between birth and 6 h of life.
c. Available chest radiography obtained within 6 h after birth.
d. Available parameters of the acid–base balance (blood pH, partial pressure of O_2_ and CO_2_, and base excess) and blood serum ionogram for Na^+^, K^+^, and Ca^2+^ evaluated in the umbilical cord blood sample.

#### 2.3.5. Exclusion criteria

The exclusion criteria are as follows:

a. Need for intubation directly after birth or perinatal asphyxia, defined as the abnormal acid–base parameters detected in an umbilical cord blood sample (pH < 7.0 or base excess ≤14 mmol/L).
b. Multiple apnea-brady that require immediate intubation prior to an NIV trial
c. Age > 24 h.
d. Meconium aspiration syndrome.
e. Air leak syndrome.
f. Congenital heart disease.
g. Congenital diaphragmatic hernia.
h. Other severe congenital malformations and genetic disorders (diagnosed before and after birth) associated with an increased risk of respiratory failure.
i. The need for surfactant administration immediately after birth, regardless of the method of administration (respiratory distress syndrome—RDS).

#### 2.3.6. Early-onset infection/pneumonia procedure

Diagnostics of early-onset infection/pneumonia follows the protocol in force. As the diagnosis of early-onset infection/pneumonia can be made later than in the first 6 h of the infant's life, patients with an infection (diagnosed on the basis a positive blood culture) or pneumonia (diagnosed on the basis of a chest x-ray) included in the study will be analysed as a separate subgroup.

#### 2.3.7. Blinded (masking)

The study team was divide between the blinded and non-blinded group. Members of the unblinded study team prepare the medicinal product/placebo (pharmacist). The remaining team members (doctors and nurses) and patiens and parents form the blinded study team.

### 2.4. Procedures and interventions

Enrolment will take place within and up to 24 h from birth (usually within the first 6 h of life). During enrolment, patients will be screened for the inclusion and exclusion criteria. Demographic data (including date and time of birth, sex, gestational age in weeks and days, and body weight at birth), obstetric history [type of delivery, single/multiple pregnancy, and prenatal steroid treatment (number of doses and date of last dose administered)], and Apgar scores will be collected, and physical examination will be conducted.

Researchers can use a variety of CPAP or NIV devices or modes, e.g., DuoPAP on FABIAN® (ACUTRONIC Medical Systems AG, Switzerland), CMV on Babylog® VN500 (Drägerwerk AG Co., Germany). Based on the available literature, it is not possible to clearly demonstrate the advantage of one of the modes of respiratory support over another in the treatment of respiratory failure in newborns. We would like the study to determine whether this is indeed the case and to what extent the change in the mode of non-invasive ventilation impacts the incidence of PPHN. Proposed CPAP initial settings: PEEP at 5–6 cm H_2_O pressure with an oxygen concentration of ≥21% to maintain pre-ductal saturation between 90% and 95%.

Patients assigned to the study group will be treated with 0.15 mg/kg body weight (diluted in 3 ml 0.9% NaCl) nebulized salbutamol (Ventolin®, GlaxoSmithKline, Dublin, Ireland) for 30 min. This dose was selected according to a previous study on salbutamol in TTN (31). The placebo group will be administered 3 ml nebulized 0.9% NaCl for 30 min.

The intervention period (during which patients will receive salbutamol or 0.9% NaCl together with NIV) will be divided into period I up to 24 h after enrolment and period II between 24 and 48 h after enrolment. Patients can be treated for up to 48 h from enrolment, with a maximum of four doses administered every 6 h. The extended follow-up period is planned between 48 h after enrolment and 7 days of life (or hospital discharge, whichever occurs first). The study design and planned examinations are shown in Figure 1.

All participants will be given drugs (IMP or placebo) by nebulization with the use of the same device—AEROGEN SOLO® (Aerogen Ltd, Galway, Ireland). This is an innovative tool which delivers aerosol *via* a vibrating mesh. Evidence suggests that the vibrating mesh nebulizer (VMN) provides a 5-fold greater aerosol delivery to the lung as compared to a jet nebulizer (JN). The nebulizer will be placed in the respiratory circuit at a short distance from the CPAP interface.

Echocardiography will be conducted at least twice during the study: (1) within 6 h after enrolment in the study and (2) 6–10 h after administration of the last dose of the drug (or placebo).

LUS will also be conducted twice during the study: (1) within 6 h after enrolment in the study, ideally simultaneously with the CXR and (2) 6–10 h after administration of the last dose of the drug (or placebo).

The modified TTN Silverman score will be determined every 1 h during the first 6 h after enrolment, every 2 h between 6 and 24 h after enrolment, and every 6 h from 24 to 48 h after enrolment.

The modified TTN Silverman score will be determined at 1 and 4 h after administration of the drug (or placebo) and at the moment of intubation (if needed). Vital signs, including respiratory rate, heart rate, fraction of inspired oxygen (FiO_2_), blood pressure (systolic, diastolic, and mean) and arterial blood oxygen saturation, will be recorded every 1 h during the first 24 h after enrolment and every 2 h between 24 and 48 h after enrolment. Additionally, vital signs will be evaluated 1 and 4 h after administration of the drug (or placebo).

### 2.5. Participants timeline

T_0_—screening and enrolment (birth to 24 h of life).
T_0_—allocation and randomization (6 to 24 h of life).
T_1_—first observation period (from randomization to 24 h after randomization).
T_2_—second observation period (from 24 to 48 h after randomization).
T_3_—extended observation period (from 48 h after randomization to discharge or 7 days of life, depending on which comes first).

### 2.6. Primary outcome

The primary outcome is the percentage of infants who develop PPHN, defined as the need for ventilation with FiO_2_ > 0.30 and features of increased pulmonary pressure on echocardiogram (patent ductus arteriosus or foramen ovale with right-to-left or bidirectional flow, as well as other echocardiographic parameters described below), and/or a > 10% difference between pre-ductal and post-ductal oxygen saturation levels.

### 2.7. Secondary outcomes

The secondary outcomes are the severity of respiratory distress (assessed with the modified TTN Silverman score, presented in Table 1) (32, 33), frequency of need for intubation, duration of NIV, duration of hospitalization, and blood levels of acid–base parameters [pH, partial pressure of CO_2_ (pCO_2_), partial pressure of O_2_, and base excess/deficit] and ions (sodium, potassium, and calcium).

**Table 1:** Modified TTN Silverman score.

	0	1	2
Saturation^a^	>95%	90%–95%	<90%
Intercostal retraction	None	Mild	Continuous
Tachypnea	None	Mild	Continuous
Nasal flaring	None	Mild	Continuous
Expiratory grunting	None	Intermittent	Continuous

TTN, transient tachypnea of the newborn.

^a^ Measurement of post-ductal saturation during oxygen therapy.

### 2.8. Measures

**Figure 2:**
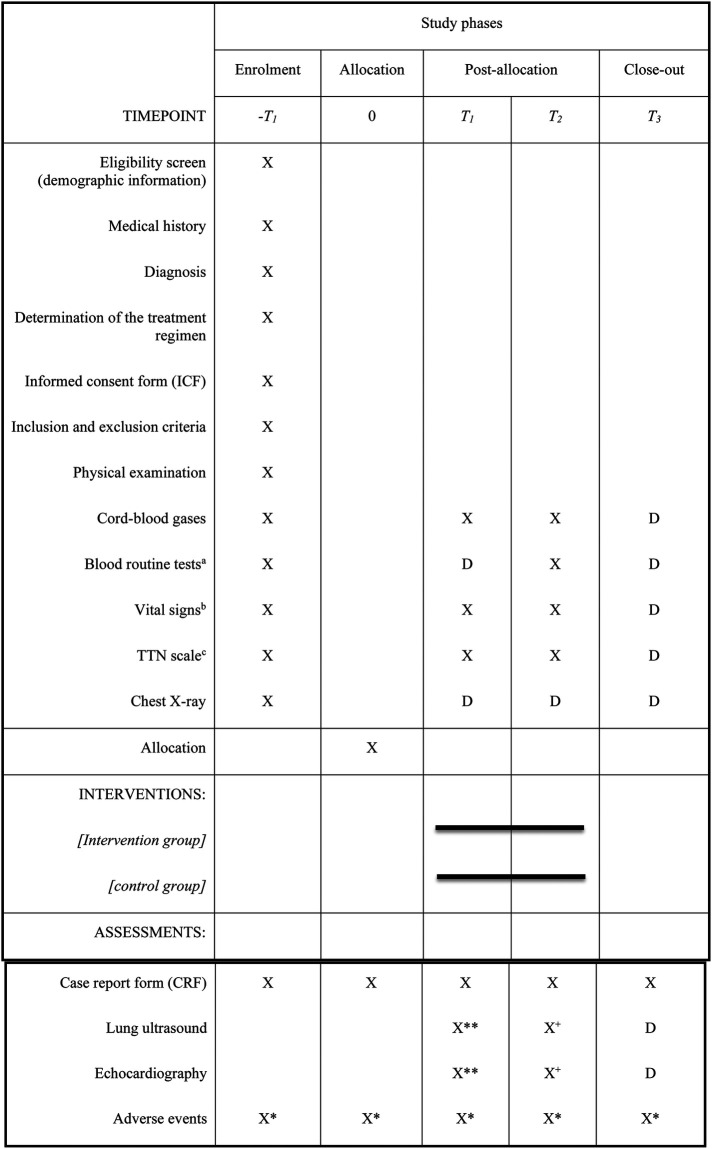
The schedule of enrolment, interventions, and assessments in the REFSAL study. D – possible performance of an additional examination according to the decision of the attending physician. Obligatory in the case of intubation, pneumothorax, or sudden deterioration of the patient's condition – procedures should be performed within 6–10 h of the patient's deterioration. ^a^Blood count, Ionogram made of venous blood, Blood gases. ^b^Every 1 h within 6 hours of randomization, every 2 h between 6 and 24 h, and every 6 h between 24 and 48 h after randomization and 1 and 4 h after the end of each dose/placebo and at the time when intubation is necessary. ^c^Every 1 h within 6 h of randomization, every 2 hours between 6 and 24 h, every 6 h between 24 and 48 h after randomization, and 1 and 4 h after the end of each dose/placebo and at the time of necessity for intubation. * Adverse events monitored from enrolment until the end of the extended follow-up period, that is, up to seven days of age or hospital discharge. **up to 6 hours after allocation. ^+^6–10 hours after the last dose of IMP.

PPHN is defined as the need for ventilation with FiO_2_ > 0.30 and features of increased pulmonary pressure on echocardiogram (patent ductus arteriosus or foramen ovale with right-to-left or bidirectional flow), and/or a > 10% difference between pre-ductal and post-ductal oxygen saturation levels. To evaluate the features of PPHN, echocardiography will be conducted at least twice (Figure 2) using a sector Doppler transducer with a frequency range of 5–12 MHz.

The examination will include the following:

a. Estimation of systolic pulmonary arterial pressure (SPAP) accounting for tricuspid valve regurgitation (using the modified Bernoulli equation) and right atrial pressure (RAP): SPAP will be estimated according to the following formula: SPAP (mmHg) = 4 × (VmaxTR)2 + RAP, where VmaxTR is the peak velocity of tricuspid regurgitation (in minutes/seconds). RAP is generally assumed to be approximately 3–5 mmHg (34).
b. Evaluation of direction and time (in minutes) of blood flow through the ductus arteriosus (right-to-left and left-to-right): This will be obtained from a parasternal short-axis view.
c. Determination of pulmonary artery flow, including measurements of right-sided systolic time intervals at the pulmonary valve in the parasternal short-axis view: This will include time to peak pulmonary artery flow velocity (TPV), right ventricular ejection time (RVET), and TPV/RVET ratio.
d. Assessment of the tricuspid annular plane systolic excursion (TAPSE): The examination will be conducted in an apical four-chamber projection using M-mode. Gating will be set near the attachment of the tricuspid valve to the myocardium.
e. Determination of the myocardial performance index (MPI, also known as the Tei index) for the right ventricle: The examination will be conducted in an apical four-chamber projection (to monitor the diastolic flow through the tricuspid valve using a pulse-wave Doppler) and in a parasternal short-axis view (to monitor the pulmonary artery flow). The isovolumetric contraction time (ICT), isovolumetric relaxation time (IRT), and ejection time (ET) will be measured, and MPI will be calculated using the formula (ICT + IRT)/ET (35, 36). The MPI will also be measured using tissue Doppler echocardiography.
f. Calculation of the eccentricity index (EI), defined as the ratio of the left ventricle dimensions (D1/D2 ratio) measured along the short axis of the heart: The examination will be conducted in a parasternal short-axis view at the level of the mitral valve.
g. Assessment of the right ventricular systolic duration to diastolic duration ratio (S/D ratio): Systolic duration is the duration from the onset to the termination of tricuspid valve regurgitation, and diastolic duration is the time between the two jets of tricuspid regurgitation. The right ventricular S/D ratio is calculated from the Doppler signal of the tricuspid valve regurgitation (34).
h. Assessment of the size of foramen ovale.

#### 2.8.1. Echocardiographic criteria for diagnosis of PPHN

1. Pulmonary arterial pressure >33% of the systemic arterial pressure.
2. Presence of right-to-left flow (that lasts >30% of the cardiac cycle) through the ductus arteriosus and/or foramen ovale.
3. TPV/RVET ratio <0.3 for mild PPHN and <0.23 for severe PPHN.
4. TAPSE <1 cm.
5. Right ventricular MPI >0.25.
6. D1/D2 ratio >0.81.

#### 2.8.2. Evaluation of TTN

The modified TTN Silverman score (32, 33) (Table 1) will be used to assess the severity of the respiratory disorder. The sum of the points will correspond to the Silverman score (0–10 range; the higher the score, the more severe the respiratory distress).

Based on Szymanski et al.'s study (13) on the evaluation of TTN, we propose to use a modified LUS score in neonates with respiratory distress, which includes posterior instead of lateral lung fields, and a five-grade rating scale, where “0” corresponded to normal lung and “4” indicated the presence of pulmonary consolidations (13). The sum of all four area scores will be the total LUS score, which will range from 0 to 16.

For NIV, the following information will be collected: start time, total duration in days, initial/maximal pressure, and initial/maximal FiO_2_.

### 2.9. Early termination

A patient can be withdrawn from the study at any stage following the decision of the principal investigator or withdrawal of consent from the parent/legal guardian. Early termination can also occur in the following cases:

• An adverse event (AE) likely related to the treatment intervention.
• The need for surfactant administration.

The need for intubation or rapid worsening of respiratory function, which is defined as follows:

a. An increase in oxygen demand by >50% for >30 min or an increase in pCO_2_ above 70 mmHg in two consecutive examinations of blood acid–base parameters performed at a 1-h interval.
b. Repeated (more than twice in 1 h) incidents of apnea lasting >20 s, defined as cessation of breathing and bradycardia <100 beats per minute with oxygen saturation <80%.
c. The need for manual ventilation using a bag valve mask.
• Improvement of respiratory function, allowing NIV discontinuation.
• Severe violation of the eligibility criteria or study protocol.

Early termination of participation in the study will result in the discontinuation of the treatment with salbutamol. After treatment discontinuation, the patient will be followed up according to the study protocol until the seventh day of life or hospital discharge.

In every case, the reason for withdrawal will be described in the electronic case report form. Patient data collected before the withdrawal can be included in the analysis by the sponsor.

### 2.10. Data management and safety monitoring

#### 2.10.1. Data collection and management

Details of data management (software, procedures, responsibilities, etc.) will be described in a data management plan prior to the trial. All medical records will be collected and stored in the web-based application “NEO-CRF,” developed and maintained by NEOVINCI—Poland. During the trial, the performance of data management and any deviations from the data management plan will be documented. Technical speciﬁcations of the trial database and all data checks will be documented in a data-validation plan. All medical records of the trial participants will be kept conﬁdential and managed under the applicable laws and regulations.

#### 2.10.2. Trial monitoring and oversight

The study will be monitored according to the monitoring plan. During monitoring visits, the study monitor will ensure that it is conducted according to the International Conference of Harmonization Good Clinical Practice guidelines and other binding standards, and verify the consistency between the content of electronic case report forms and the source data defined in the monitoring plan to an extent. Additionally, the data will be periodically overseen by an individual (designated by the sponsor) responsible for data quality control. All questionable entries will be resolved by the investigator and documented properly.

The sponsor is entitled to perform an audit in the participating centers and sites. The audit will be conducted according to a predetermined plan. Appropriate authorities (local and/or international) can inspect the participating sites any time. During an audit or inspection, the investigator is obliged to provide the controllers with full source documentation and all electronic case report forms collected during the study.

#### 2.10.3. Safety monitoring

Safety will be determined for all patients who receive salbutamol or placebo, according to the procedures described earlier in this section. Patients will be analyzed according to the actual treatment received. All data regarding safety from enrolment to the end of the follow-up period (seventh day of life or hospital discharge) will be reported and analyzed. AEs and serious AEs (SAEs) will be categorized according to the newest version of the MedDRA System Organ Class and Preferred Term classification. The severity of AEs and their relationship with treatment will also be reported.

## 3. Discussion

PPHN affects up to 6.8 per 1,000 term or near-term neonates (11). Despite progress in the management of PPHN, the mortality rate is approximately 4%–33% (11). The expected short-term health benefits of salbutamol include a shorter duration of NIV, reduced oxygen concentration in the respiratory gas mixture, and an improved respiratory function according to the modified TTN Silverman scale. The expected long-term health benefits of salbutamol include a reduced risk of PPHN and, consequently, a reduced risk of severe cardiorespiratory failure requiring intensive care (including iNO and ECMO), reduced mortality risk, and reduced risk of long-term outcomes such as cerebral palsy. As previous studies have not reported any safety issues for the treatment of neonates with nebulized salbutamol, it can be concluded that this treatment is likely to be beneficial to this patient group (18, 23, 32). Nevertheless, our study has its limitations that can influence its outcomes. A considerable limitation is associated with the difficult differential diagnosis of TTN and respiratory distress syndrome in neonates with young gestational age. Another one is regarding the definition of pulmonary hypertension that may also bring some that can suggest this disorder but cannot fully diagnose it.

## Data Availability

The original contributions presented in the study are included in the article/Supplementary Material, further inquiries can be directed to the corresponding author.

## The REFSAL study group

1. Department of Neonatology and Neonatal Intensive Care, Medical University of Warsaw, Warsaw, Poland
2. Clinical Department of Neonatology with Neonatal Intensive Care Unit, Saint Jadwiga the Queen Clinical Provincial Hospital No. 2, University of Rzeszow, Rzeszow, Poland
3. Department of Neonatology, Collegium Medicum in Bydgoszcz Nicolaus Copernicus University, Toruń, Poland
4. Neonatology Clinic, University Hospital, Kraków, Poland
5. Department of Neonatology and Neonatal Intensive Care., Pomeranian Medical University, Szczecin, Poland
6. Neonatology Clinical Department Karol Marcinkowski University Hospital, Zielona Góra, Poland
7. Neonatology Unit, Specialist Hospital No 2, Bytom, Poland
8. Department of Neonatology, Poznan University of Medical Sciences, Poznan, Poland

**Consulting partners**:

1. Department of Pediatrics-Division of Neonatology, Faculty of Medicine, University of Ottawa, Ottawa, Canada
2. Department of Cardiac and General Pediatric Surgery, Medical University of Warsaw, Poland
3. Pediatric Radiology Department, Medical University of Warsaw, Poland

**The REFSAL Study Group**: WB (Clinical Department of Neonatology with Neonatal Intensive Care Unit, Saint Jadwiga the Queen Clinical Provincial Hospital No 2, Rzeszow, Poland)—witekblaz@yahoo.com, RB—PI (Department of Neonatology and Neonatal Intensive Care, Medical University of Warsaw, Warsaw, Poland)-E-mail: renata.bokiniec@wum.edu.pl, MB-K (Neonatal and Intensive Care Department, Medical University of Warsaw, Warsaw, Poland)—maria.borszewskakornacka@gmail.com, MB (Pediatric Radiology Department, Medical University of Warsaw)—mbrzewski@me.com, TD (Department of Pediatrics-Division of Neonatology, Faculty of Medicine, University of Ottawa, Ottawa, Canada) tdaboval@cheo.on.ca, Emanuela Ferretti (Department of Pediatrics-Division of Neonatology, Faculty of Medicine, University of Ottawa, Ottawa, Canada)—eferretti@toh.ca, Piotr Korbal and IS-K (Department of Neonatology, Collegium Medicum in Bydgoszcz Nicolaus Copernicus University, Toruń, Poland)—pkorbal@cm.umk.pl; sadowskakrawczenko@gmail.com, Agnieszka Kijanka (Department of Neonatology and Neonatal Intensive Care Medical University of Warsaw, Warsaw, Poland)—a.kijanka@vp.pl, Jacek Kuźma (Department and Cardiac and General Pediatric Surgery, Medical University of Warsaw)—kuzmajacek@yahoo.com, RL and Agnieszka Ochoda-Mazur (Neonatology Clinic, University Hospital, Kraków, Poland)—ryszard@lauterbach.pl; a.ochoda@gmail.com, BŁ (Department of Neonatology and Intensive Neonatal Care Pomeranian Medical University, Szczecin, Poland)—beatal@pum.edu.pl, DM (Neonatal and Intensive Care Department, Medical University of Warsaw, Warsaw, Poland)—dariusz.madajczak@wum.edu.pl, MM-K (Neonatology Clinical Department)—marzena.michalak.kloc@gmail.com, Karol Marcinkowski University Hospital, Zielona Góra, Poland), Joanna Seliga-Siwecka (Department of Neonatology and Neonatal Intensive Care, Medical University of Warsaw, Warsaw, Poland)—joanna.seliga@wum.edu.pl, HS (Neonatology Unit, Specialist Hospital No 2, Bytom, Poland)—helen_s@autograf.pl, TS (Department of Neonatology, Poznan University of Medical Sciences, Poznan, Poland)—tszczapa@gmail.com, and Marta Szymankiewicz (Department of Neonatology, Gynecology and Obstetrics Clinical Hospital, Poznan University of Medical Sciences, Poznan, Poland)—mszymanek@ump.edu.pl.
